# Transcriptional Regulation of Genes by Ikaros Tumor Suppressor in Acute Lymphoblastic Leukemia

**DOI:** 10.3390/ijms21041377

**Published:** 2020-02-18

**Authors:** Pavan Kumar Dhanyamraju, Soumya Iyer, Gayle Smink, Yevgeniya Bamme, Preeti Bhadauria, Jonathon L Payne, Elanora Dovat, Morgann Klink, Yali Ding

**Affiliations:** Department of Pediatrics, Pennsylvania State University College of Medicine, Hershey, PA 17033, USA; pdhanyamraju@pennstatehealth.psu.edu (P.K.D.); chidambaram.soumya@gmail.com (S.I.); gsmink@pennstatehealth.psu.edu (G.S.); ybamme@pennstatehealth.psu.edu (Y.B.); preetibhadauria66@gmail.com (P.B.); jpayne2@pennstatehealth.psu.edu (J.L.P.); edovat@pennstatehealth.psu.edu (E.D.); mreed7@pennstatehealth.psu.edu (M.K.)

**Keywords:** Ikaros, tumor suppressor, gene transcription, leukemia

## Abstract

Regulation of oncogenic gene expression by transcription factors that function as tumor suppressors is one of the major mechanisms that regulate leukemogenesis. Understanding this complex process is essential for explaining the pathogenesis of leukemia as well as developing targeted therapies. Here, we provide an overview of the role of Ikaros tumor suppressor and its role in regulation of gene transcription in acute leukemia. Ikaros (IKZF1) is a DNA-binding protein that functions as a master regulator of hematopoiesis and the immune system, as well as a tumor suppressor in acute lymphoblastic leukemia (ALL). Genetic alteration or functional inactivation of Ikaros results in the development of high-risk leukemia. Ikaros binds to the specific consensus binding motif at upstream regulatory elements of its target genes, recruits chromatin-remodeling complexes and activates or represses transcription via chromatin remodeling. Over the last twenty years, a large number of Ikaros target genes have been identified, and the role of Ikaros in the regulation of their expression provided insight into the mechanisms of Ikaros tumor suppressor function in leukemia. Here we summarize the role of Ikaros in the regulation of the expression of the genes whose function is critical for cellular proliferation, development, and progression of acute lymphoblastic leukemia.

## 1. Clinical Significance of Ikaros Activity in Leukemia 

Acute lymphoblastic leukemia (ALL) is the most commonly encountered malignancy in childhood with a long-term cure rate approaching 90% [1]. Although cure rates have steadily improved over the last 60 years, there continue to be subsets of patients with a poor prognosis [2]. Mutations or deletions of *IKZF1* have shown to have a poor prognosis in precursor B-cell acute lymphoblastic leukemia (B-ALL) [2,3,4,5]. *IKZF1* is a gene that encodes the Ikaros transcription factor that helps regulate genes controlling cell cycle progression and cell survival [2,3,4,5]. Ikaros is one of the major regulators of normal hematopoiesis, and is required for all lymphoid lineage development. Ikaros knock-out mice lack B and T lymphocytes and natural killer cells, as well as their defined progenitors [6]. *IKZF1*-inactivating mutations and/or deletions are seen more commonly in patients with other poor prognostic features including elevated white blood cell count, an age over 10 years old, and the Philadelphia chromosome. B-ALL patients with *IKZF1* abnormalities have a reduced 5-year event free survival of 61% compared to the 87% for those without this abnormality. *IKZF1* mutations and deletions are more commonly seen in B precursor ALL compared to T precursor ALL [7]. *IKZF1* genetic alterations occur both in childhood and adult B-ALL. ALL is the most common pediatric malignancy, and about 60% of ALL cases occur in patients that are younger than 20 years old. In adults, ALL represents only 20% of all acute leukemias, but it has much worse prognosis as compared to pediatric ALL. It was reported that approximately 50% of adult patients have *IKZF1* genetic alterations, including over 80% of patients with BCR-ABL1 positive (Ph+) ALL [8]. *IKZF1* genetic changes are seen in approximately 15% of childhood B-cell ALL, including up to 70% of patients with BCR-ABL1 positive (Ph+) ALL [2,3,4,7,9]. Another subset of ALL is Ph-like ALL, which exhibits a genetic profile similar to Ph+ ALL. Ph-like ALL represents 15%-20% of cases and has been shown to have inferior outcomes compared to other precursor B-ALLs [10]. A majority of individuals that have a Ph-like phenotype have also been found to have *IKZF1* deletions to various degrees. Patients with *IKZF1* deletions have been shown to have higher rates of induction failure (7% *vs* 1%, *p* = 0.009), leading to poorer outcomes [5]. Although cure rates for pediatric ALL continue to improve, relapse leads to significant pediatric mortality [2,11,12]. Patients with *IKZF1* deletions have also been found to have an increased risk of relapse and a reduction in overall survival [3,5,13]. Those individuals with *IKZF1* deletions treated according to standard therapy had a 12-fold increased risk of relapse [13]. In a study where *IKZF1* deletion was used to risk stratify and intensify therapy, patients with B-ALL had improved outcomes [14]. This shows the promise of using *IKZF1* to further risk stratify patients with B-ALL with the hopes of reducing relapse and improving long-term cures. The role of *IKZF1* is less understood in T-cell ALL. *IKZF1* mutations have been shown to play a role in up to 5% of T-ALL and as high as 11% of early T cell precursor (ETP) ALL [15]. *IKZF1* genetic and functional abnormalities were also studied and considered as novel prognostic biomarkers for high-risk leukemia in several clinical trials (NCT00993538; NCT03709719, NCT01431664).

## 2. Ikaros as a Transcription Factor and Epigenetic Regulation of Its Target Genes

**Figure 1:**
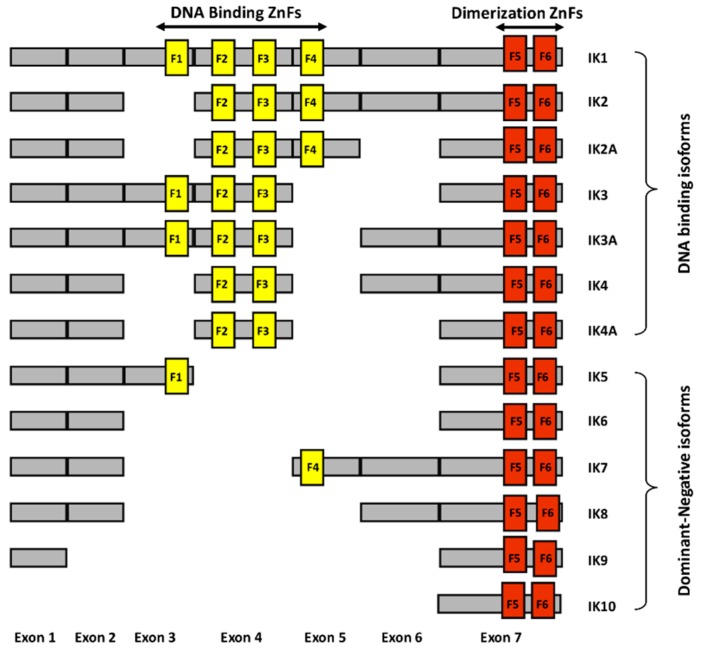
Schematic diagram of different human Ikaros isoforms. The N-terminal zinc fingers (F1–F4) are shown in yellow vertical bars and C-terminal zinc fingers (F5–F6) are shown in orange vertical bars.

Ikaros is a zinc finger protein with N-terminal DNA binding domains and C-terminal dimerization domains [16,17]. The full length of Ikaros has four N-terminal zinc fingers which are involved in DNA binding, and two C-terminal zinc fingers which are involved in protein-protein interactions (Figure 1.). Ikaros has various isoforms with conserved C-terminal dimerization domains but with a different number of N-terminal zinc fingers [18]. Isoforms lacking N-terminal zinc fingers do not bind DNA and can act as dominant negatives [19]. Ikaros appears to function both as a transcriptional repressor and as an activator through its ability to bind to different nuclear factors involved in epigenetic regulation and chromatin remodeling. If it binds to histone deacetylase complexes, it causes gene repression. If it binds to ATP-dependent chromatin remodeling complexes SW1/SNF, it causes gene activation.

Ikaros binds to the promoter regions of its target genes, repressing gene expression by formation of repressive chromatin through two distinct mechanisms [20,21]. Direct Ikaros binding to the promoters without HDAC1 results in increased H3K9me3 and reduced H3K9ac, while Ikaros recruitment of HDAC1 results in increased H3K27me3 and reduced H3K9ac [21]. It was also reported that Ikaros can activate enhancers and super-enhancers as well as possess pioneering activity by opening closed chromatin, which would result in the activation of its target gene expression [22]. Thus, Ikaros can regulate expression of its target genes both directly, by binding to their promoters, as well as via altering the global epigenetic signature of enhancer and super-enhancer landscapes. Global genome-wide binding experiments were performed by several research groups aiming at identification of Ikaros target genes in different cell types. Though numerous potential Ikaros target genes emerged in the high-throughput sequencing data, the potential role of Ikaros in direct regulation of expression of these genes has not been studied. Understanding the mechanisms about how Ikaros regulates these target genes and their role in leukemia will help in discovering new therapeutic targets in leukemia. Here, we review the Ikaros role in regulation of its target genes, whose function is important for the development and/or progression of leukemia. 

## 3. Phosphatidylinositol-3 Kinase (PI3K) Pathway 

The phosphatidylinositol-3 kinase (PI3K) signaling network is a highly regulated pathway important for various physiological processes. This pathway has been implicated in various diseases, such as cancer, as well as in resistance to cancer due to its aberrant activation [23]. Dysregulation of the PI3K pathway has also been shown in various leukemias, making this pathway a prime target for therapeutic intervention [15,16,17,18,19,20,21,22,23]. Several PI3K pathway inhibitors are being tested as potential anti-cancer agents. Hence, the understanding of the regulation of the PI3K pathway in leukemia cells is important. Song et al. (2015) demonstrated the importance of Ikaros and how it modulates the proliferation of leukemic cells by inhibiting the transcription of the genes involved in the PI3K pathway [24]. Authors used chromatin immunoprecipitation followed by deep-sequencing (ChIP-Seq) to map Ikaros binding to gene promoter sites in a B-ALL cell line, Nalm6 and in primary B-ALL cells. Ikaros binding sites in the promoter regions of the genes which regulate the PI3K pathway were identified. Several genes were identified as Ikaros target genes, including those that promote and inhibit the PI3K pathway (e.g., PIP4K2A, PIKFYVE, INPP5D). Ikaros was shown to suppress transcription of genes that promote the PI3K pathway (most notably oncogene PIK3CD), with the exception of the phosphatase INPP5D (SHIP1), which is positively regulated. Furthermore, regulation of each of these genes was systematically investigated in this study using loss-of-function and gain-of-function experiments. These experiments demonstrated that overexpression of Ikaros resulted in repression of transcription of genes that promote the PI3K pathway, but induced transcription of a gene that inhibits the PI3K pathway (INPP5D). Overexpression of Ikaros resulted in negative regulation of the PI3K pathway, as evidenced by reduced phosphorylation of AKT, a downstream target of the PI3K pathway. These data demonstrated the critical role of Ikaros in regulation of the PI3K pathway in leukemia. 

## 4. Cell Cycle Pathway

The cell cycle is a highly orchestrated process regulated by cyclins and cyclin-dependent kinases (CDKs). These proteins play crucial roles in differentiation, apoptosis and epigenetic regulation. Genetic defects in CDKs cause cancers including leukemia, and pharmacological inhibition of CDKs has become a therapeutic option [25]. A study highlighting the importance of Ikaros in regulating the proliferation of leukemic cells in B-ALL was shown by Song et al. (2015) [24]. The results show that Ikaros modulates proliferation of leukemic cells by suppressing the transcription of cell cycle progression genes. ChIP-seq analysis showed that Ikaros binds to the promoter region of genes important in progression of the cell cycle. qChIP analysis was used to confirm Ikaros occupancy of target genes. ANAPC1, ANAPC7, CDK2, CDK6, CDC2, CDC7, CDC16, CDC25c, CDC25a, CCND3, and CCNE2 are among several clinically significant cell cycle progression genes identified in Nalm6, as well as in primary B-ALL samples. Overexpression of Ikaros in Nalm6 led to a decreased expression of genes that modulate cell cycle progression compared to cells transduced with empty vector. This was associated with partial cell cycle arrest. Overexpression of Ikaros also directly repressed promoter activity of these genes (as evidenced by luciferase reporter assay). An opposite effect was noted in human B-cell acute lymphoblastic leukemia cell line—Nalm6 cells following transfection with Ikaros shRNA. Thus, Ikaros knockdown led to increase in transcription of the cell-cycle-promoting genes. These changes in the function and expression of Ikaros target genes due to gain or loss of Ikaros presents evidence that Ikaros negatively modulates cell cycle progression. Specific inhibitors of several Ikaros target genes that promote cell cycle progression (e.g., CDK2) have been developed and tested for targeted therapy for malignant diseases. In leukemia, tumor suppressor functions of Ikaros are impaired and therefore not sufficient enough to regulate Ikaros target gene transcription to the extent required for halting leukemic cell proliferation. Thus, these results illustrate the importance of Ikaros in modulating cell cycle progression in B-ALL. 

## 5. Lysine-Specific Demethylase 5B (KDM5B)

KDM5B, also known as JARID1B, is a histone lysine demethylase involved in demethylation of histone 3 lysine 4 (H3K4). It has been shown to be mutated and overexpressed in several tumors [26,27,28]. It is involved in tumor initiation, infiltration, and metastasis [29,30]. KDM5B modulates the expression of tumor suppressors and oncogenes by regulating the methylation levels of H3K4 in cancer cells [28,31]. A study by Wang et al. (2015) used high risk B-ALL cells to investigate regulation of this important global epigenetic regulator, KDM5B [32]. Increased expression of KDM5B was observed in B-ALL cells compared to normal bone marrow. Overexpression of Ikaros via retroviral transduction resulted in repression of the KDM5B gene in both B-ALL and T-ALL cells. Conversely, knock-down of Ikaros with shRNA resulted in increased expression of KDM5B. DNA-binding studies identified that Histone Deacetylase 1 (HDAC1) is involved in transcriptional repression of KDM5B. Molecular and pharmacological targeting of HDAC1 demonstrated that HDAC1 is essential for Ikaros-mediated transcriptional regulation of KDM5B. Analysis of the epigenetic signature at KDM5B promoter revealed that Ikaros regulates KDM5B expression by recruiting HDAC1 to the promoter of the KDM5B gene, induces a repressive chromatin state, and causes subsequent transcriptional repression. These results suggest that Ikaros mediates its tumor-suppressive activity and global regulation of gene expression in leukemia by negatively regulating the expression of KDM5B. 

## 6. Plant Homeodomain Finger 2 (PHF2)

Plant homeodomain finger 2 (PHF2) belongs to the Jumonji C (JmjC) class of proteins and encompasses a JmjC domain and plant homeodomain finger. PHF2 is involved in the demethylation of H3K9me2 [33]. PHF2 is a positive epigenetic modulator and is linked to tumor suppression in various kinds of cancer. PHF2 aids in differentiation of tumor-initiating cells [34]. The expression of PHF2 is markedly reduced in various subsets of acute lymphoblastic leukemia (ALL) patients. Low expression levels of PHF2 correspond to proliferation of leukemic cells and are a poor prognostic marker in B-ALL [35]. Ge et al. (2018) identified PHF2 as a direct target of Ikaros [35]. The results demonstrated that patients with B-ALL that carry deletion of a single copy of *IKZF1* have significantly lower levels of PHF2 and that Ikaros positively regulates expression of PHF2. Biochemical experiments showed that expression of PHF2 is upregulated by Ikaros via chromatin remodeling, as evidenced by increased H3K4me3 occupancy at the promoter of the PHF2 gene. Overall, results of this study showed that PHF2 is downregulated in ALL, and that deletion and/or functional inactivation of *IKZF1* might be one of the reasons for low levels of PHF2 in high-risk ALL. Low levels of PHF2 in combination with Ikaros deletion are the possible markers of high-risk ALL. 

## 7. Cytokine Receptor-like Factor 2 (CRLF2)

Cytokine receptor-like factor 2 (CRLF2) is an IL-7 like cytokine. It plays a crucial role during normal hematopoiesis [36,37,38,39]. Genetic alterations leading to overexpression of CRLF2 have been shown to be associated with pediatric acute lymphoblastic leukemia (ALL) [40,41,42,43]. *IKZF1* deletions have been observed in 43% of pediatric ALL with overexpression of CRLF2 [44]. Of late, several studies have shown that Ikaros modulates the expression of its target genes via remodeling of chromatin in ALL [21,24,45,46]. Ge et al. (2016) demonstrated strong Ikaros binding in the CRLF2 promoter region in B-ALL cells [47]. In high-risk leukemia, the expression of CRLF2 was elevated and correlated with poor clinical outcomes. The functional experiments showed that Ikaros binds to the promoter region of CRLF2 and suppresses its expression in ALL cells by altering epigenetic signature at the CRLF2 promoter. Results suggest that inhibition of CRLF2 expression is one of the mechanisms through which Ikaros exerts its tumor-suppressive effects in ALL. This study was the first to show that deletion of *IKZF1* may be one of the reasons for elevated levels of CRLF2 in high-risk ALL with no CRLF2 rearrangement. Elevated levels of CRLF2 might co-operate with *IKZF1* deletion, driving oncogenesis in ALL. The study demonstrated for the first time the existence of an IKZF1-CRLF2 pathway in high-risk ALL and suggested that targeting this pathway could be used as a therapeutic approach for high-risk B-ALL.

## 8. AT-Rich Interaction Domain 5B (ARID5B)

One of the most commonly dysregulated families of DNA-binding factors across multiple cancers is the AT-rich interactive domain (ARID) family [48,49]. The ARID proteins interact with DNA [50] and encompass the *Jumonji (jmj)* domain which plays a crucial role in histone modification and transcription. An important member of the ARID family of proteins is ARID5B, which recognizes the core DNA motif AAT(C/T) [51] and plays a cardinal role in differentiation and growth of B-cell progenitors [52]. Studies have demonstrated that ARID5B interacts with histone deacetylases (HDACs) and PHD finger protein 2 (PHF2), a histone demethylase [53,54,55]. Of late, several studies addressing the genome-wide association of *ARID5B* have shown that single nucleotide polymorphisms (SNPs) within *ARID5B* are critically affiliated with high-risk B-ALL [56,57]. Furthermore, abnormal expression of ARID5B pauses maturation of B-cells in the developing fetus and adds to leukemogenesis [58]. Multiple studies have suggested that *IKZF1* and *ARID5B* SNPs are positively correlated with ALL [59,60,61,62,63,64,65,66,67,68,69,70,71], but studies showing the connection between ARID5B expression and *ARID5B* SNPs were missing. Ge et al. (2018) demonstrated that the deletion of a single copy of *IKZF1* correlates with low expression of ARID5B and that ARID5B expression is positively regulated by Ikaros [72]. The results show the correlation of *IKZF1* and *ARID5B* SNPs with increased risk of ALL and suggest that alterations in SNPs may be associated with ALL due to reduced expression of Ikaros and ARID5B. Overall, the data suggest that the oncogenesis of high-risk ALL involves low levels of ARID5B expression, and that low expression of ARID5B and PHF2, along with haploinsufficiency of Ikaros, represents a high-risk subgroup of ALL.

## 9. c-MYC and MYC Binding Protein 2 (MYCBP2)

c-myc is a well-known oncogene whose overexpression is associated with various types of malignancies, including leukemia and lymphoma. MYC binding protein 2 (MYCBP2), a member of the PHR family of proteins is an E3 ubiquitin ligase [73]. Increased levels of MYCBP2 are originally found in the axon guidance and synapse formation in the nervous system [73,74]. The activation or inhibition of various signaling pathways is enhanced by ubiquitination ligase activity of MYCBP2. Pathways regulated by ubiquitin ligase activity of MYCBP2 include inhibition of the p38 MAPK and IL-10 signaling pathway and activation of the mTOR pathway. Mechanisms independent of ubiquitin ligase activity include regulation of Rheb where MYCBP2 acts as a guanosine exchange factor (GEF) [75,76,77]. Additionally, the binding of MYCBP2 to the MYC protein facilitates the transcriptional activation of MYCBP2 [78]. In Burkitt’s and AIDS-associated lymphomas, this region is frequently mutated, signifying that MYCBP2 suppresses MYC activity [73]. The decreased expression of MYCBP2 has been detected in both B- and T-ALL patients. High expression of c-MYC along with reduced expression of MYCBP2 were observed in adult ALL patients and correlated with liver infiltration, splenomegaly, and worse clinical course [45]. Sequence analysis of the promoter regions of both c-myc and MYCBP2 in adult ALL patients revealed strong Ikaros binding sites [45]. Gain- and loss-of-function studies of Ikaros in ALL showed that Ikaros represses transcription of the c-myc gene, but positively regulates transcription of MYCBP2 [45]. Adult patients with ALL who had haploinsufficiency of IKZF1 exhibited increased levels of c-myc and reduced levels of MYCBP2. These data suggest that Ikaros can affect cellular proliferation in ALL by regulation of c-myc and MYCBP2 expression. 

## 10. B-cell Lymphoma 6 (BCL6) and BTB and CNC Homology 1 Basic Leucine Zipper Transcription Factor 2 (BACH2)

B-cell Lymphoma 6 (BCL6) is a proto-oncogene primarily identified in diffuse large B-cell lymphomas (DLBCLs) [79,80]. BCL6 has a role in germinal center formation and in antibody maturation [81]. Moreover, BCL6 is a zinc finger transcriptional repressor targeting the downregulation of over 500 genes mainly involved in the cell cycle, gene transcription, resistance to DNA damage, and regulation of chromatin structure [82]. 

The increased expression of BCL6 regulates the protection and maintenance of leukemia stem cells and the survival of pre B-cells [83]. In ALL and CML, BCL6 attenuates the survival of leukemic cells from chemotherapy-induced DNA damage through repressing p53 and Arf and by inducing FOXO3a signaling [83,84]. Furthermore, BCL6 potentiates the sensitivity of B-ALL patients to methotrexate by elevating ZEB1 expression [85]. In ALL cells, increased expression of BCL6 results in resistance to DNA damage which subsequently increases survival during BCR-ABL1 kinase inhibition [86]. Finally, B lymphoblasts with BCR-ABL phenotype lacking BCL6 were not able to induce leukemia in immunodeficient mice [86]. 

BTB and CNC Homology 1 Basic Leucine Zipper Transcription Factor 2 (BACH2) is a transcriptional factor associated with the germinal center formation and affinity maturation of B-cells [87,88,89,90]. It functions as a tumor suppressor and pre B-cell receptor checkpoint in pre B-ALL, CML, and Ph-positive ALL cells and induces apoptosis in response to oxidative stress [91,92,93]. Clinically, loss of BACH2 levels corresponds with lower disease-free survival in pediatric ALL patients [94,95,96]. Mutations like deletions or loss of heterozygosity of BACH2 locus attributes to 30% of pre B-ALL cases [97,98]. In leukemia and lymphoma, BCL6 orchestrates BACH2 protein stability and the balance of the BCL6/BACH2 axis is essential in regulating pre B-cell receptor checkpoint cascades [99]. 

Recent studies showed that *IKZF1* deletion is associated with high BCL6 and low BACH2 expression in adult B-ALL patients [100]. Strong Ikaros binding to promoters of both BCL6 and BACH2 was observed. Functional experiments demonstrated that Ikaros represses transcription of BCL6 and activates transcription of the BACH2 gene. These data suggest that one of the mechanisms of tumor-suppressor activity of Ikaros in ALL involves regulation of the BCL6/BACH2 axis. 

## 11. Dynamin 2 (DNM2)

Dynamin 2 (DNM2) regulates a variety of cellular processes including intracellular vesicle formation and trafficking, receptor endocytosis, interactions of actin and microtubule, cytokinesis, cell invasion and migration, and regulation of apoptosis [101,102,103,104]. The DNM2 protein has five domains: GTPase domain; intermediate domain (MD); pleckstrin homology domain (PH); GTPase effector domain (GED); and proline-arginine-rich domain (PRD). Recurrent mutations in all DNM2 domains including the GTPase domain have been linked to T-ALL [105,106,107,108,109]. It has been suggested that DNM2 plays a major role in the internalization of IL7R, TCR, and Notch ligand Delta like 1 (DIl-1), thereby triggering the development of ALL [110]. Recently, somatic mutations of *DNM2* were identified in pro-B ALL, with all of the mutant variants centric to the middle domain [111]. Loss of functional mutations in DNM2 has resulted in the increase of IL7R cell surface expression in a Lmo2 transgenic T-ALL mouse model [112]. 

Ikaros binding to the promoter of DNM2 was observed in both B-ALL and T-ALL cells [108]. Overexpression of Ikaros in both types of leukemia resulted in reduced transcription of DNM2. This was associated with enrichment of the H3K9me3 epigenetic marker at the DNM2 promoter. Ikaros knockdown resulted in increased expression of DNM2. These data suggested that Ikaros represses DNM2 in leukemia via direct binding to the DNM2 promoter and by inducing formation of heterochromatin.

## 12. Interleukin-7 Receptor-α (IL7R) and SH2B Adaptor Protein 3 (SH2B3)

IL7R is responsible for the differentiation of hematopoietic cells into lymphoid progenitor cells, and its level is altered during the development of T- and B-cells [113,114]. The IL7R gene encoding the IL7R-α chain heterodimerizes with the IL7R-γ form or with the cytokine receptor-like factor 2 (CRLF2) to form IL7 receptor or thymic stromal lymphopoietin (TSLP) receptor, respectively [115,116,117]. Coupling of the receptor chains results in the phosphorylation of tyrosine residues on the receptor leading to the activation of downstream JAK/STAT and PI3K/Akt/mTOR signaling cascades [118,119,120,121,122,123]. In leukemia, somatic mutations in IL7R are prevalent in 10% of pediatric T-ALL cases and gain-of-function mutations are reported in both T-ALL and B-ALL [124,125]. Within the B-ALL molecular subtypes, IL7R expression values were higher in the BCR/ABL1, ETV6/RUNX1, TCF3/PBX1, and KMT2A(MLL) high-risk leukemia subtypes [123]. 

The SH2B adaptor protein 3 (SH2B3), also known as lymphocyte adaptor protein (LNK), is a key negative regulator of the cytokine and tyrosine kinase signaling pathways, thus playing a crucial role in hematopoiesis [126,127,128]. The SH2B3 protein specifically binds to phosphorylated tyrosine and acts as a central negative regulatory node for multiple signaling pathways [86,129]. Loss-of-function mutations in SH2B3 are reported to play an important role in the oncogenesis of myeloproliferative neoplasms (MPN), early T-ALL, Ph-like ALL, B-ALL and nonmalignant hematological diseases by accelerating STAT3 phosphorylation and increased NOTCH-1 signaling [130,131,132,133]. Thus, SH2B3 can attenuate IL7-stimulated JAK/STAT5 signaling.

Recently, a subtype of adult B-ALL that has high expression of IL7R coupled with low expression of SH2B3 has been identified and found to be associated with a more severe clinical picture and poor prognosis [134]. Further analysis of this patient population revealed that high expression of IL7R coupled with the low expression of SH2B3 is strongly associated with haploinsufficiency of *IKZF1* due to deletion of one *IKZF1* allele. Analysis of genome-wide occupancy in B-ALL showed that Ikaros binds to the promoters of both the IL7R and SH2B3. Gain-of-function and loss-of-function experiments demonstrated that Ikaros negatively regulates expression of IL7R and activates transcription of the SH2B3. Ikaros binding to the promoter of the SH2B3 is associated with enrichment in H3K4me3 occupancy. These data suggest that Ikaros directly regulates expression of IL7R and SH2B3.

Overall, the above-summarized data show that Ikaros plays a highly important and potentially a critical role in regulation of IL7/JAK/STAT5 signaling by directly regulating transcription of at least three genes that are a critical part of this signaling pathway: CRLF2, IL7R and SH2B3.

## 13. Conclusions

**Figure 2:**
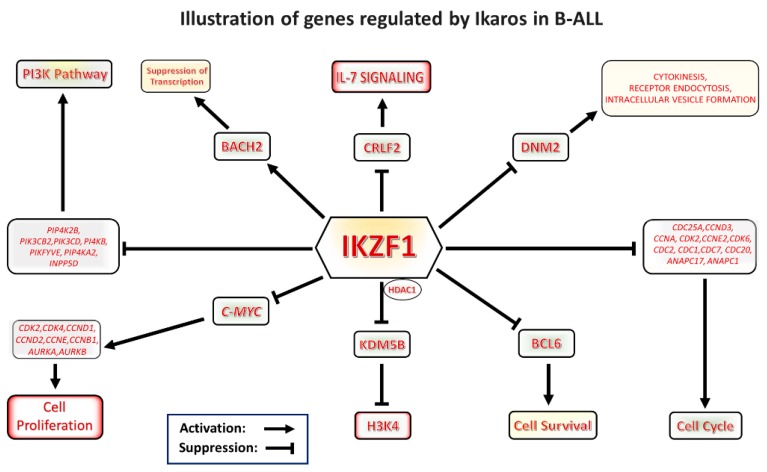
Graphical representation of Ikaros signaling in B-cell acute lymphoblastic leukemia (B-ALL). Ikaros is a master regulator and tumor suppressor which modulates transcription of a number of genes important for leukemogenesis. The drawing illustrates various pathways regulated by Ikaros.

In summary, Ikaros is a potent transcription factor that regulates expression of a large number of genes in normal hematopoietic cells and in leukemia. Since Ikaros target genes have diverse roles in various cellular activities, Ikaros plays important roles in regulation of multiple signaling pathways and cellular functions via regulation of expression of its target genes (Figure 2.) Some of Ikaros’ target genes are critical components of the same signaling pathway (e.g., IL7R, CRLF2 and SH2B3 in the IL7R/JAK/STAT5 signaling pathway; multiple cell-cycle-promoting genes in cell cycle progression; or the PI3K pathway). Because of this, these pathways are strongly affected by Ikaros activity, and Ikaros most likely exerts its function as a tumor suppressor in leukemia via control of these particular signaling networks. Targeting Ikaros-regulated signaling pathways could be a highly effective therapeutic approach in high-risk leukemias that are characterized by Ikaros genetic inactivation (*IKZF1* haploinsufficiency due to deletion of one *IKZF1* allele). Identification of other Ikaros target genes would be important, both to gain insight into Ikaros function as a tumor suppressor, as well as to identify additional therapeutic targets for high-risk acute lymphoblastic leukemia.
